# Fabricating ordered 2-D nano-structured arrays using nanosphere lithography

**DOI:** 10.1016/j.mex.2017.07.001

**Published:** 2017-07-19

**Authors:** Chenlong Zhang, Sandra Cvetanovic, Joshua M. Pearce

**Affiliations:** a Department of Materials Science & Engineering, Michigan Technological University, Houghton, MI, USA; b Department of Electrical & Computer Engineering, Michigan Technological University, Houghton, MI, USA

**Keywords:** Microsphere lithography, Plasmonic, Nanosphere lithography, Dip coating, Spin coating, Nanosphere, Plasmonic, Metamaterial, Photovoltaic, Synthesis

## Abstract

Recent advances in the use of plasmonic metamaterials to improve absorption of light in thin-film solar photovoltaic devices has created a demand for a scalable method of patterning large areas with metal nanostructures deposited in an ordered array. This article describes two methods of fabricating ordered 2D nanosphere colloidal films: spin coating and interface coating. The two methods are compared and parameter optimization discussed. The study reveals that:

• For smaller nanosphere sizes, spin coating is more favorable, while for larger nanospheres, the angled interface coating provides more coverage and uniformity.
• A surfactant-free approach for interface coating is developed to fabricate zero-contamination colloidal films.
• Each of the methods reaches an overall coverage of more than 90% and can be used for nanosphere lithography to form plasmonic metamaterials.

## Graphical abstract

**Figure:**
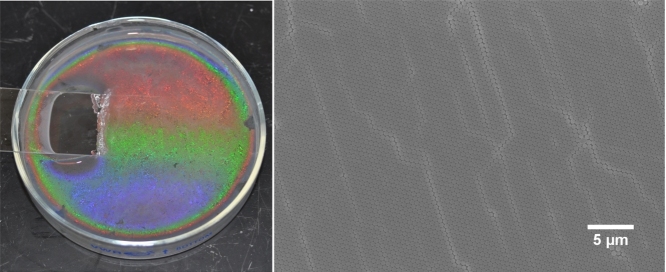

## Method details

There is substantial interest in the use of plasmonic metal nanostructures to form metamaterials for improving light absorption in thin-film solar photovoltaic (PV) devices [1], [2]. Sophisticated light management in thin-film solar PV devices has become increasingly important in that they ensure absorption of the entire solar spectrum while reducing semiconductor absorber layer thicknesses, which in turn reduces deposition time, material use, embodied energy and greenhouse gas emissions, and economic costs [2]. Metal nanostructures have a strong interaction with light, which enables unprecedented control over the propagation and the trapping of light in the absorber layer of thin-film PV [3], [4], [5]. This has created a demand for a scalable method of patterning large areas with metal nanostructures deposited in an ordered array. Common methods to fabricate such arrays (e.g. e-beam lithography) are expensive and not practical for such large areas. Nanosphere lithography has been considered an alternative way of fabricating scalable plasmonic arrays [6] in an inexpensive and scalable fashion. With care in subsequent etching and evaporation processes, geometries from simple triangle arrays to more complex structures such as rings, dots, and rods can be fabricated [7]. In the past two decades several nanosphere coating techniques have been developed to acquire nanosphere masks, including spin coating [8], [9], dip coating [10], and interface coating [11], all aimed at attaining high order uniformity and fewer defects. Spin coating is most common at the lab-scale due to its high efficiency in producing self-organized particle monolayers, as well as its flexibility in controlling the process, allowing sophisticated manipulation on colloidal crystal geometry, double- or multi-layer colloidal crystals, and even non-closed packed crystals [12], [13]. However, the spin coating process is not simple as it involves fine tuning several parameters, which have interdependent effects on the evaporation process. Finding these parameters is an art, largely dominated by empiricism. For researchers who want to use spin coating in their nanosphere lithography related research they often have to develop their own recipes, and generally the optimal recipe varies depending on sphere size [14].

Interface coating, also known as the Langmuir-Blodgett method, refers to the process of forming a monolayer on the liquid-air interface, which is then transferred to a solid substrate. With the assistance of surfactants [11], 2-D colloidal spheres self-assemble into monolayer domains. Interface coating is attractive to industry because of its insensitivity to substrate materials and relative ease of implementation. However, additional processes like surface modification are often necessary to acquire well-ordered patterns [15].

Using 500 nm and 1000 nm polystyrene nanospheres, this article compares the two methods in detail and proposes two novel and convenient recipes for both nanosphere sizes. The hexagonal close-packed (HCP) coverage is determined from the scanning electron microscopy (SEM) and quantified with the free and open-source image-processing software ImageJ (https://imagej.nih.gov/ij/). The results show two methods to obtain >90% surface coverage with a defect-free close-packed domain area up to 1 mm^2^. Additionally, this is the first time an interface coating method has been described that does not require any additional surfactants or surface modification. The plasmonic 2-D silver nanotriangle arrays are fabricated in subsequent steps and their size tuned by annealing the substrate in dry nitrogen flow.

## Close-packed sphere lithography

### Spin coating

Spin coating involves dropping a colloidal suspension on a hydrophilic substrate, followed by an accelerated evaporation process in a spin coater. Several parameters affect the spin coating process such as the spin velocity and acceleration, the size and concentration of the nanospheres, the substrate wettability, and ambient pressure and humidity. In applications involving the use of spin coating for nanosphere lithography, the goal is to form large-scale, well-ordered arrays. For decades, researchers have had to empirically find the best recipes for their own applications since the published recipes have so far been largely non-reproducible by others [16]. Chen et al. illustrate the mechanism of the spin coating evaporation process and develop recipes by mapping the spin and acceleration speeds for various nanosphere sizes [14]. A recent survey on nanosphere-related publications, however, reveals that researchers who use spin coating are still randomly developing empirical protocols, and working on a theory [17], [18]. Colson et al. did a statistical analysis on published spin coating parameters and then predicted those parameters using a mathematical model [16]. The study reported a 200 μm^2^ defect-free domain using the parameters provided by their model [16]. It must be noted, however, that the success of the model is built on 490 nm nanospheres, which is inapplicable in the majority of cases. In this study, 500 nm and 1000 nm nanospheres in aqueous suspension were used for spin coating at various speeds in order to observe the differences in the process depending on small or large bead sizes. This study is not aimed at developing a general recipe, but instead aims to show the trend of changing parameters, and, more importantly, to compare spin coating with the alternative methodology of interface coating. This could act as a useful guideline for researches in many applications.

For spin coating polystyrene nanospheres, the following steps are used:

1. A 6 inch (∼154 mm) silicon wafer was cut into 1 inch by 1 inch (25 mm × 25 mm) pieces.
2. The c-Si substrates are cleaned using a modified RCA solution (H_2_O_2_:NH_4_OH:H_2_O = 1:1:5) at 110 °C for 40 min. The solution oxidizes organic residuals and renders the surface hydrophilic.
3. Cleaned wafer substrates are kept in deionized water before use.
4. The substrate is dried under nitrogen flow and then transferred to the spin coater sample holder.
5. Polystyrene nanospheres 500 nm and 1000 nm in diameter are purchased from Fisher Scientific Inc.
6. The nanosphere suspension is centrifuged at 7500 rpm for 10 min, which disperses the suspension into solutions with different water/ethanol ratios.
7. The solution undergoes ultrasonication for 1 h in order to ensure that the beads in the suspension are uniformly dispersed throughout the suspension and not clumped together.
8. A 200 μL nanosphere suspension is dropped onto the substrate and allowed to expand freely in order to cover the entire surface of the substrate for 2 min.
9. Spin coating with a pre-set rotation velocity and acceleration is used. The initial rotation velocity and acceleration is 1500/600 (rotation/acceleration) and 3000/1750 for the 500 nm and 1000 nm nanospheres respectively. The rotation velocity is increased in subsequent tests while the acceleration speed is kept constant.
10. The rotation duration is varied from 2 to 5 min depending on the solvent evaporation rate.
11. Coated colloidal crystal masks are dried in air and stored in a desiccator.

After the completion of the array synthesis, a morphology analysis is carried out using the JEOL S4700 field emission scanning electron microscope (FE-SEM). The masks for FE-SEM analysis are coated with 2 nm platinum to prevent direct exposure to the electron beam.

Spin coating of polystyrene spheres with diameters less than 500 nm has been widely studied and the recipes well established. The theoretical studies by Denkov et al. and Zhao & Marshal [17], [18] show that inter-particle capillary forces are the driving forces for the ordering process. Capillary forces arise as a result of the increasing curvatures of the liquid surface between particles. As evaporation of the solvent continues, capillary forces squeeze the nanospheres into a crystal domain since the nanospheres seek the lowest energy configuration and consequently tend to maximize contact with neighbors, leading to a HCP structure. The process continues with flux from the border to compensate for the evaporated liquid, supplying more nanospheres to the domain until all the liquid has evaporated. In this way, the spheres self-assemble. When spin coating under high speeds, the suspension is spun away from the center, leaving the central part less wet. The ordering hence starts from the center and extends towards the exterior, a phenomenon confirmed through observation of the spreading white ring from center to border during the spin coating process.

**Fig. 1:**
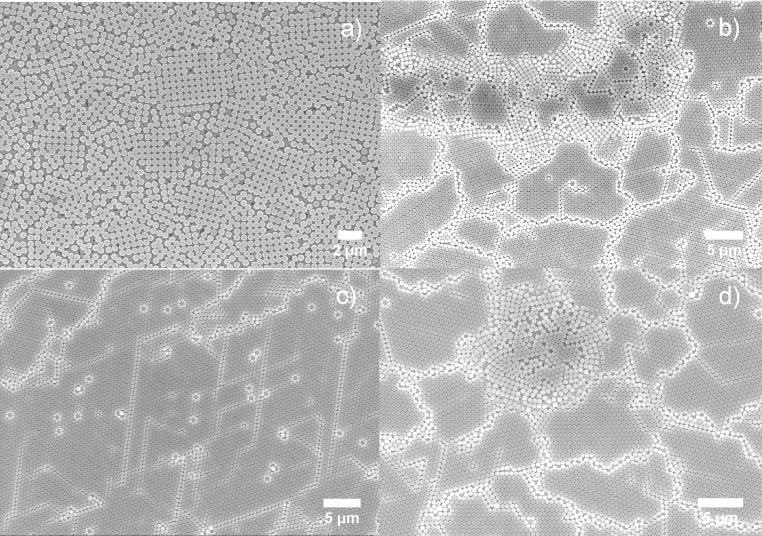
SEM images of polystyrene 500 nm nanospheres in 10 wt% aqueous solution spin coated on an Si (100) wafer at rotation speed = 1500 rpm (a), 3000 rpm (b), 6000 rpm (c) and 10,500 rpm (d), acceleration speed = 600 rpm/s (a–d).

Past studies have shown that the high evaporation rate is the key to yielding high HCP coverage, which in turn requires faster spinning speeds during the spin coating process [19]. The results presented here are in agreement with these theoretical conclusions. As can be seen in Fig. 1a–c, increasing the rotation speed minimizes the bilayer coverage and yields a more ordered HCP. At a rotation speed of 6000 rpm, the bilayer disappears, leaving the whole surface covered by the HCP. The calculated HCP coverage is ∼98%, with only a tiny area at the corner uncovered. Further increasing the rotation speed does not further increase the HCP coverage − on the contrary, small bilayer clusters were observed at center of the structure. The emergence of these clusters is attributed to the rapid evaporation rate of the solvent while the radial centrifuge force is relatively weak at center. Spheres on top have therefore not yet had a chance to be spun out before the solvent evaporates. Irregularity in non-close-packed, bilayer, and multilayer structures are often found in fast evaporation systems, such as the water/ethanol system [13].

**Fig. 2:**
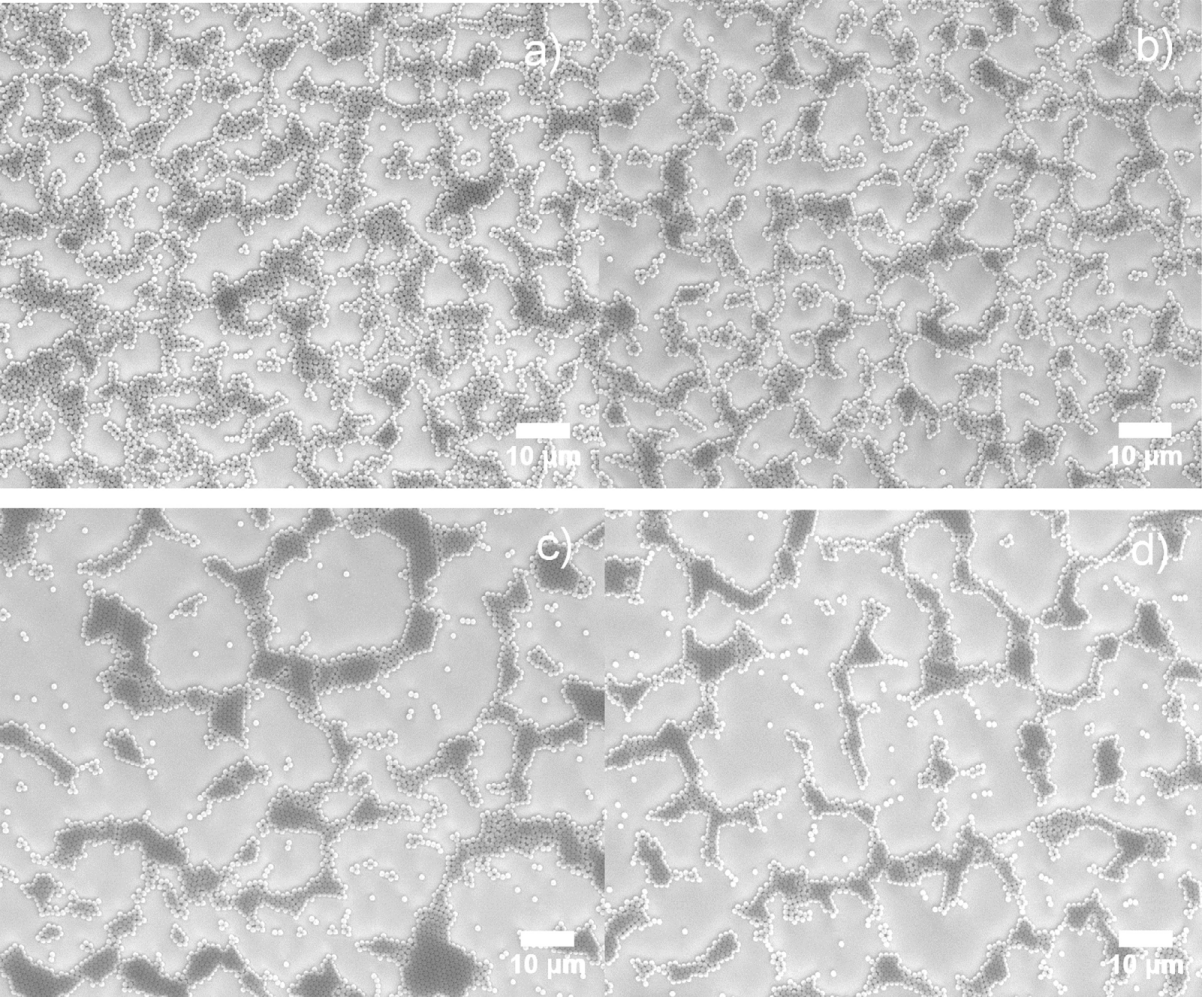
SEM images of polystyrene 1000 nm nanospheres in 10% wt aqueous solution spin coated on Si (100) wafer at rotation speed = 2000 rpm (a), 4000 rpm (b), 6000 rpm (c) and 8000 rpm (d), acceleration speed = 1500 rpm/s (a–d).

**Fig. 3:**
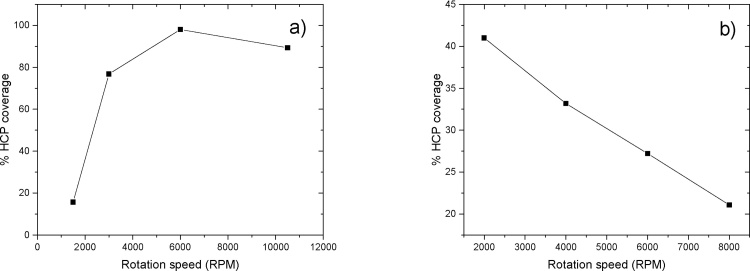
500 nm (a) and 1000 nm (b) rotation speed vs HCP coverage.

Spin coating on nanospheres with diameters of 1000 nm or above have seldom been reported successfully. Chen et al. report that their optimum settings for 1300 nm PS nanospheres are about 4000 rpm in rotation speed and 1600 rpm/s in acceleration [14]. However, their 1300 nm sample has noticeable voids when compared to other samples with PS beads of less than 510 nm [14]. In this study, similar results were found (Fig. 2). As the rotation speed increases, the beads form discrete irregular HCP domains with the presence of free individual beads and bead clusters increasing. The overall HCP coverage decreases with the rotation speed. According to theory [17], the continuous ordering process relies on two major factors: 1) the capillary forces due to inter-particle liquid evaporation, which pushes beads together, and 2) water flux compensation, which supplies more beads to the ordered domain, so that the domain grows. The SEM analysis reveals that the inter-particle capillary force are weakened in low speed samples, as the beads are loosely attached to each other and there is no long range HCP, the force is strengthened as the rotation speed increases, as a result, more close-packed structure forms gives arise to the dark area on SEM images (Fig. 2c, d). Moreover, the overall coverage reduced with rotation speed, indicating the diameter of the bead plays an important role in HCP formation. According to Denkov et al. [17], the onset of ordering process starts earlier and the evaporation-induced horizontal capillary forces in the central area draw suspension flux from the boarder aggressively while the centrifuge force is pushing beads away from center. As a consequence, there is insufficient compensation flux and less sphere supplies to the ordered domain. This results in more voids added to the ordered area. The speed influence on small beads, like those 500 nm in diameter, can be generalized in Fig. 3a. Contrawise, high rotation speed facilitating the evaporation process creates more and larger voids on the surface for large nanospheres (Fig. 3b).

**Fig. 4:**
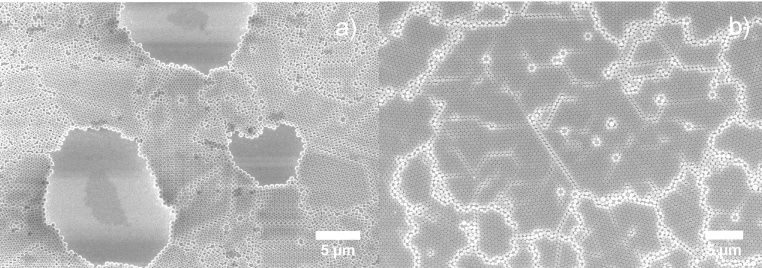
SEM images of nanosphere (diameter = 500 nm) spin coated on glass surface (a) and silicon surface (b) at rotation speed = 8000 rpm, acceleration speed = 600 rpm/s.

On the other hand, increasing the mass of the beads increases the fiction between beads and the wafer surface. Once the beads are attached to the surface due to the increased fiction, larger beads are immobilized before they can reach and join the ordered domain, thus increasing unordered areas. This mechanism is also supported by investigating smaller beads deposited on rough surfaces. Fig. 4 shows 500 nm beads on soda lime glass surface (a) and on polished silicon surface (b), both coated using the same recipe. The voids and disorders on the soda lime glass surface are similar to those found in 1000 nm silicon samples. By speeding up the evaporation, the horizontal sucking capillary force increases, which is a good for smaller beads as they move faster toward the ordered domain, but for larger beads the increase in the evaporation rate also increases the vertical component of the capillary force, which presses them against the glass surface. The increasing friction between the beads and the surface immobilizes a greater number of beads and thus results in more voids in the colloidal mask.

### Angled interface coating

Interface coating refers to the process of coating nanospheres at the interface of two media, usually air and water. This process is also known as Langmuir-Blodgett coating. The ordered nanosphere array forms due to the free-assembly of the nanospheres at the interface. The array can be subsequently transferred to a substrate surface. Interface coating is easy to implement and, unlike evaporation processes such as spin coating that demand fine-tuned parameters, there are fewer variables that need to be precisely adjusted to achieve high uniformity and a well-ordered monolayer. This process also excludes the formation of bilayer assemblies and, unlike the results shown above for spin coating, it is relatively insensitive to substrate morphology and hence various substrates with hydrophilic surfaces can be used. Interface coating does however require careful manipulation of the surface tension and solvent pH, with the withdrawal angle and velocity being critical paramters when producing high quality colloidal film. In the past, these protocols demanded additional modification of the solvent with chemicals, such as surfactants [20], alkaline [11], and other devices or tools [21]. Introducing a surfactant is widely accepted to help acquire larger areas of ordered colloidal films with considerable mechanical strength, since surfactant molecules occupy the interface and push the incoming beads together, ceasing the Brownian motion of individual bead or small bead clusters. This forces them to join and form larger domains, increasing monolayer order [20]. Surface tension at the interface is largely reduced due to the presence of the surfactant, which in turn facilitates bead movement along the interface to find their lowest energy configuration. The result is a more ordered HCP monolayer [22]. Adding the surfactant, however, introduces contamination to the interface. These contaminates can be transferred to substrate in the lift-up process and are especially difficult to remove, thus creating imperfections in the nanosphere lithography.

**Fig. 5:**
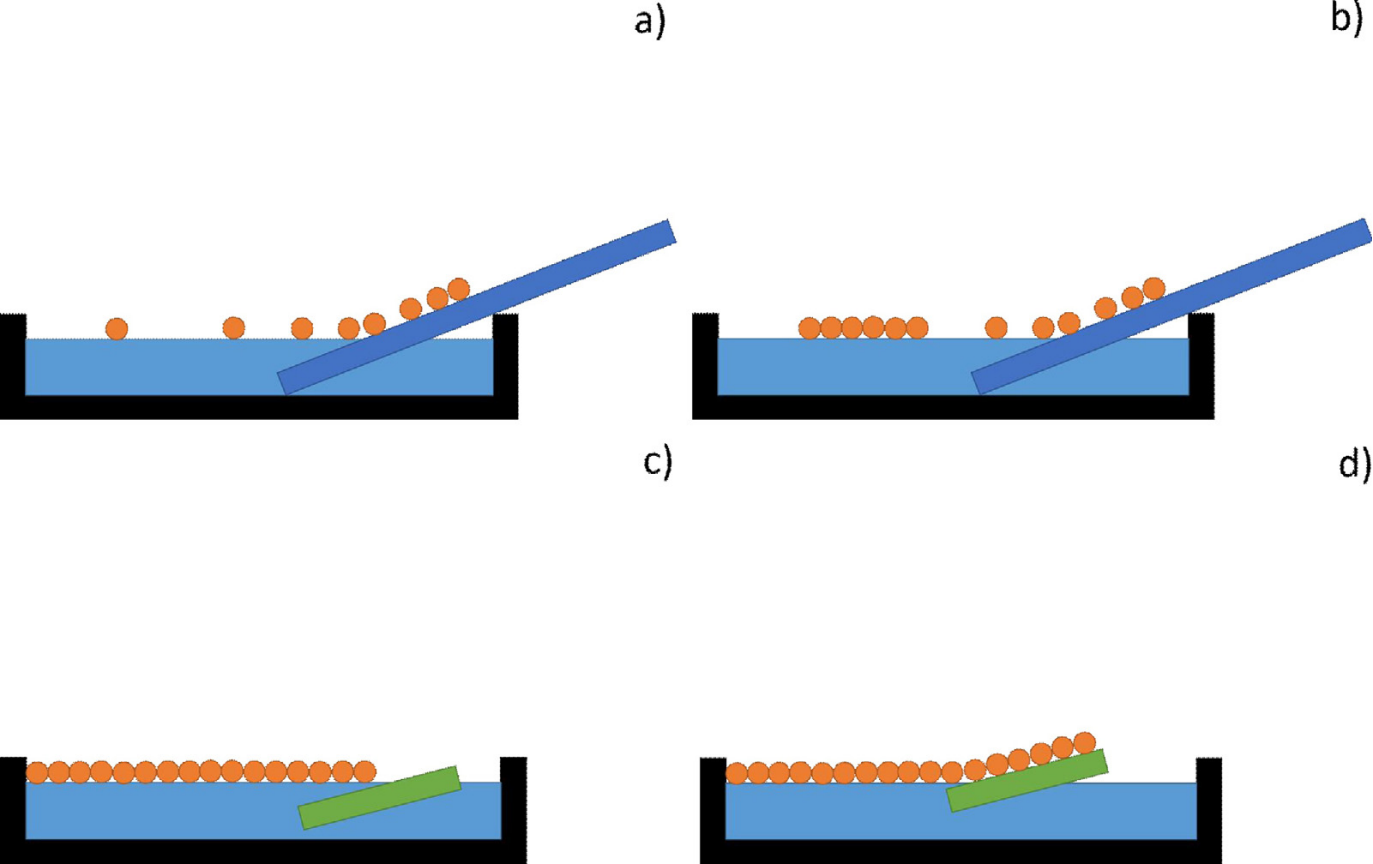
Schematics of interface coating. (a) Nanosphere suspension pipetted onto an angled glass substrate and move onto water/air interface. (b) Nanosphere start assembling on water interface. (c) As more suspension join the monolayer, a full coverage over the surface is obtained. (d) Substrate entered the water phase at a shallow angle (∼10°) to transfer the monolayer.

This study presents a convenient method to fabricate large scale arrays using angled interface coating without using any surfactant (Fig. 5a–d):

1. The nanosphere suspension is centrifuged at 7500 rpm for 10 min
2. It is then redispersed in a solvent (H_2_O/ethanol, v/v = 1:1) to have 10% solid weight.
3. For substrates, microscopic glass slides were purchased from VWR Corp.
4. The glass substrates are cleaned in piranha solution (98% H_2_SO_4_ and 30% H_2_O_2_, v/v = 3:1) at 100 °C for 30 min. It should be noted, that this process can be scaled to larger pieces of glass using the same approach.
5. The cleaned glass slides are stored in deionized water for no more than a week before use.
6. At room temperature, the microscopic glass substrate is dried under N_2_.
7. A glass Petri dish is filled with deionized water.
8. The glass slide is positioned at a 45° angle in the Petri dish as shown in Fig. 5a. This angle is chosen since it optimizes that speed at which the suspension droplet enters the water/air interface.
9. 20 μL of the newly made nanosphere suspension is pipetted onto the glass slides and moves freely along the slides into the water/air interface. It should be noted that each time only a tiny drop (∼2 μL) is pipetted, with the next drop not being added to the glass slide until the prior drop has completely diffused and there is no visible movement identifiable by naked eye on the water/air interface. By doing so, the new drop will not interfere with the diffusion process of the prior drop and turbidity is thus minimized. It should also be noted that the pipetting can be done by hand or automated with an open source syringe pump [23].
10. Pipetting the suspension is continued at a constant speed until the entire interface is covered by colorful gratings caused by the diffraction from the ordered nanosphere array.
11. The array is transferred to a 1′ by 1′ hydrophilic silicon or glass substrate at an angle of 10° with respect to the water/air interface using a 3-D printed wafer holder as shown in Fig. 6. The merit of 3-D printing is that one can design and fabricate labware in a fast and easy way [24], [25]. Any form of fused filament fabrication RepRap 3-D printer is capable of printing these wafer holders.

**Fig. 6:**
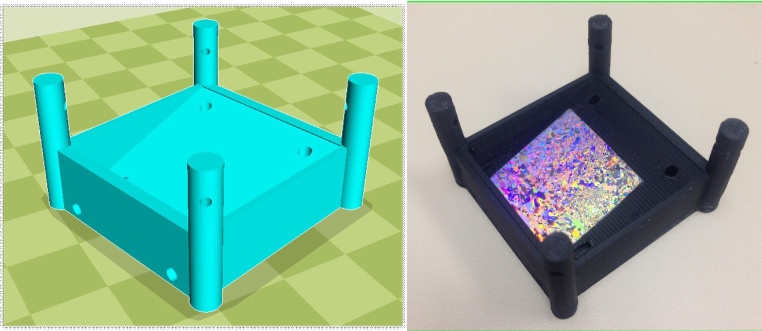
a) Rendered OpenSCAD file as an STL file in the open source slicer Cura showing design of angled wafer holder for 3-D printing, b) a printed holder holding a wafer on angled surface.
12. Finally, the lifted wafers are air dried − placed in a 3-D printed holder so that they form an angle with the ground, ensuring that all the water is optimally evaporated. They are then ready to be used for any application (e.g. as a mask for plasmonic enhancement of photovoltaic devices [26]).

To demonstrate and test this methodology, the samples are analyzed in FE-SEM after depositing 2 nm of platinum, which is sputtered in order to provide good conductivity for FE-SEM analysis.

**Fig. 7:**
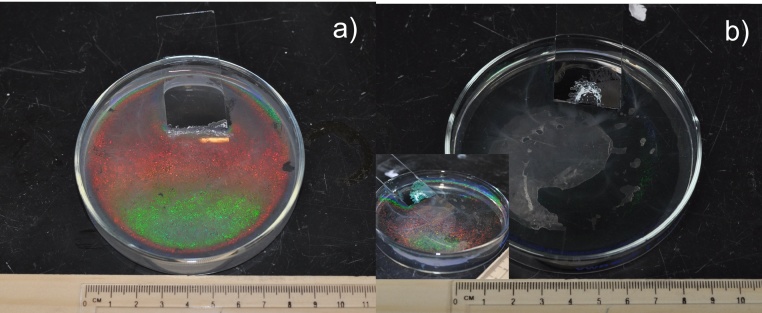
Full coverage of nanosphere monolayer on water surface without using SDS, nanosphere diameter = 1000 nm (a) and 500 nm (b). The photos were taken using digital camera with built-in flash on. Due to the optical property difference, the 500 nm nanosphere sample does not show diffraction at the same angle of observation as the 1000 nm nanosphere does. The inset image of (b) shows the photo of the same sample taken at a different angle.

Uniform coverage of monolayer beads is obtained by gently adding the beads suspension to the glass dish through angled slide. Beads that are free for diffusion across interface require time to become stable. Quickly pipetting the beads suspension results in clusters or agglomerates appearing as white residues in the water phase. In the initial state, the beads suspension droplets at the edge of the angled glass slide gradually release the beads into the water/air interface. The 1000 nm beads slide onto the interface without aggressive diffusion, instead forming a uniform monolayer at the moment they engage the water. The process is similar to that of making an omelet where the whisked egg slowly poured onto a hot pan immediately forms a flat crust. In contrast, the 500 nm beads diffuse rigorously and soon reach the border of the glass dish. In addition, some of the 500 nm beads rush into the water phase and cause an optically white suspension. With the assistance of ethanol, the lighter beads, the faster they move. This results in more freestanding beads at the interface and suspending beads in the water phase. For the 500 nm beads, no noticeable monolayer forms at the beginning, but when more bead suspension is added to the water phase, the beads start to form scattered monolayer domains. The area of these domains increases as they receive new beads and join with other proximal domains. Towards the end of the pipetting stage, full surface coverage can be achieved (Fig. 7). In both the 500 nm and 1000 nm bead cases, full surface coverage can be achieved by continuously pipetting the beads suspension until white turbidity can be observed by naked eyes, indicating that there’s no more room on the interface for new beads to join the monolayer.

To provide a contrast between surfactant-aid and surfactant-free coating, the interface coating is divided into two groups: in Group A, no surfactant is added and the colloidal coating is transferred to the substrates 30 min after full coverage has been identified by naked eye; in Group B, a 2 wt% anion surfactant, sodium dodecyl sulfate (SDS), is added to the self-assembled monolayer. It is observed that the monolayer is rigorously pushed when the surfactant solution is pipetted into the interface. SDS molecules occupy the interface aggressively, and the colloidal film is pushed away to leave room for the SDS. For full surface coverage, since introducing SDS does not push the colloidal film much, it is believed the nanospheres on a fully covered surface have already formed the close-packed structure and their mechanical properties are strong enough to resist the spreading SDS modules.

**Fig. 8:**
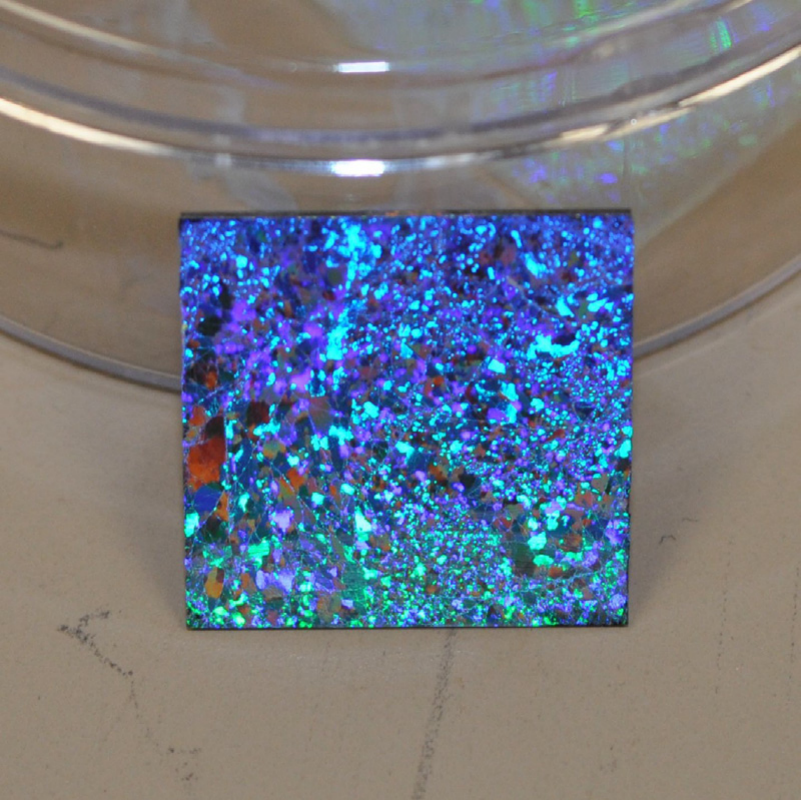
Transferred colloidal monolayer (1000 nm nanospheres) on silicon substrate (1′ × 1′). Observed area of single crystalline block reaches 3 mm^2^.

**Fig. 9:**
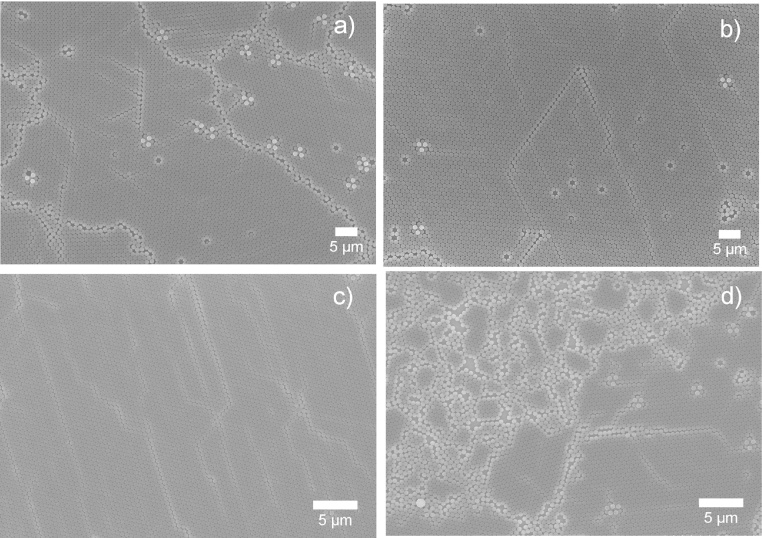
1000 nm (a,b) and 500 nm (c,d) nanosphere monolayer via interface coating with (a,c) and without (b,d) the assistant of 2 wt% SDS.

Fig. 8 shows 1000 nm of colloidal film being transferred onto a silicon substrate using a surfactant-free recipe. The 2-D crystalline blocks can be easily distinguished by their distinct diffraction orientations, which lead to different colorations at the same angle of observation. Large and uniform diffraction regions indicate large, ordered crystalline domains. The largest domain observed here is about 3 mm^2^, which seldom reported by other groups. For 1000 nm colloidal films, FE-SEM analysis barely shows any difference between Group A and Group B samples except that Group A has a slightly higher content of bead triplets due to the stress release mechanism when two crystalline domains join (Fig. 9a, b). For the 500 nm samples, SEM analysis reveals the importance of using a surfactant. With the assistance of SDS, a uniform and a well-ordered HCP structure is obtained with interface coating (Fig. 9c, d). Fewer triplets and dislocations are found in the SDS samples. In SDS-free samples, significant voids and unordered domains and large numbers of triplets and vacancies are observed.

**Fig. 10:**
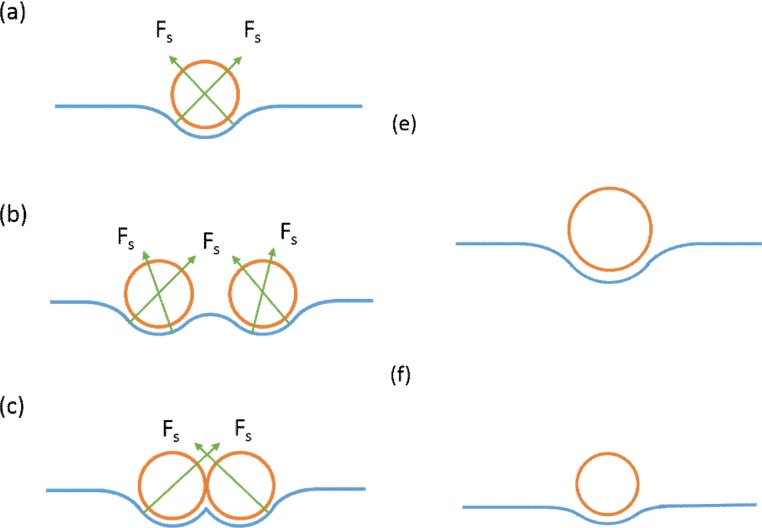
(a) An individual bead is balanced by the surface force. (b) When another beads is approaching, the balance of the horizontal component of the surface force is broken and mutual attraction of the two beads is demanded to re-balance the force. (c) The two beads stick to each other and the force is rebalanced. (e–f) Unlike larger beads, smaller beads need less surface force to balance its weight and therefore less likely to attract free nearby beads. They undergo Brownian motion until there is less room left and the interaction with surrounding beads increases. This process occurs when the whole surface reaches full coverage.

**Fig. 11:**
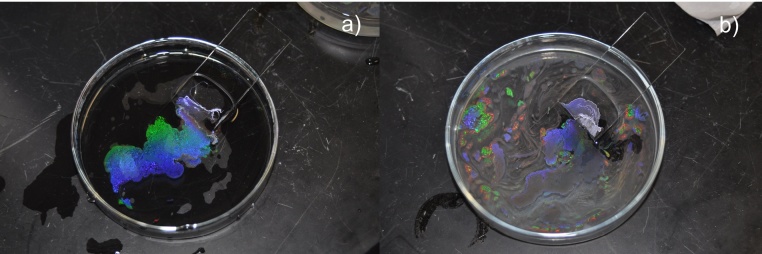
Digital images show result of colloidal film made from pipetting (a) 5 droplets of beads suspension, each contains ∼1 μL suspension and (b) 1 droplet of 5 μL suspension.

**Fig. 12:**
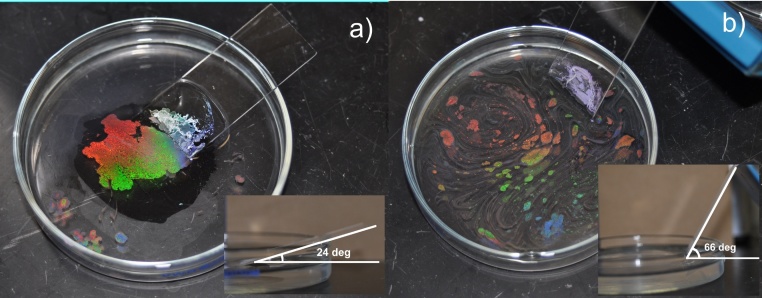
Colloidal film made from pipetting ∼2 μL 1000 nm nanospehre suspension at (a) 24° and (b) 66° angled glass slides.

A possible explanation for this observation is that for larger beads, the mutual attraction is greater. In order to balance the bead weight, the water’s surface must be curved in order to provide a surface tension force with a large enough vertical component to support the beads (Fig. 10a). The larger the beads, the greater the supporting force, and hence a more curved water surface is induced, which leads to larger areas in which the beads can interact with other beads. When a bead is caught in Brownian motion, the curvature captures nearby beads and forms a small cluster to balance the horizontal component of the surface tension (Fig. 10b, c). The clusters in turn capture more beads, and the process repeats itself to form a uniform, close-packed monolayer in hexagonal symmetry. This process can be analogously understood by imagining throwing a volleyball on a large flat sheet of fabric. The bending of the sheet caused by the weight of one volleyball draws other volleyball nearby. This process repeats itself as long as there are new beads join the monolayer domain. Introducing the surfactant is not necessary. It is worth mentioning that the process is strongly influenced by the size of the droplets as well as the angle at which the assisting glass slide is placed, both of which control the initial speed of the droplet as it engages the water/air interface. Fig. 11, Fig. 12 show the angle and droplet size effects. The larger the droplets or slope, the faster the engaging speed, leading to an increase in turbidity and a more scattered crystal domain (Figs. 11 b, 12 b). Even though the scattered domains may join into larger domain eventually, the domain boundary density increases, introducing more defects to the film. When using smaller droplets (∼1 μL) and inserting them at shallow angles, the beads are much more likely to form a crystalline monolayer immediately after engaging the water/air surface (Figs. 11 A, 12 a). Continuously adding the beads in this manner will only increase the area of the monolayer. It is good practice to produce high quality film by pipetting the smallest droplet on a slide that is at a shallow angle (e.g. around 24°).

If the bead weight is too small, the induced surface tension may not be able to bend the water surface enough to draw in the nearby beads (Fig. 10e, f). The beads remain in Brownian motion until the surface tension is modified by other processes (e.g. by introducing a surfactant to the interface). At the same temperature, smaller particles additionally tend to move faster and are less easily captured by other beads or bead clusters. Consequently, voids and vacancies are found more often in 500 nm bead samples without SDS. During film transfer, it is also found that for 500 nm Group A samples, the monolayer is fragile and breaks apart when trying to transfer it onto a substrate. This is due to the weaker attractions between the smaller beads, which makes it more difficult for them to hold their positions during transfer. The Group B samples shows stronger mechanical strength, and the film is easily lifted up from the interface with no broken parts found. For the 1000 nm beads samples, both groups display similar mechanical strength and both are easily lifted up, proving that a strong interaction exists among larger beads even without the aid of a surfactant. It can therefore be concluded that for smaller beads, using a surfactant is required in order to achieve high HCP coverage.

Bilayers, voids, and non-HCP structures are considered defects and are not added to HCP percentage, which are summarized in Table 1. This selection of method and optimization depending on nanosphere size will help facilitate the process and production of contamination-free samples for fabrication and research.

**Table 1:** Comparison of monolayer coverage by fabrication methods and bead size.

	500 nm spin coating	1000 nm spin coating	500 nm interface coating (SDS)	1000 nm interface coating (SDS)	500 nm interface coating (no SDS)	1000 nm interface coating (no SDS)
HCP%	98	72	90	91	54	89

## Conclusions

In this study, novel approaches have been demonstrated to fabricating large colloidal HCP structures using polystyrene beads. Spin coating was found to be the more favorable way of implement coating for smaller (500 nm) beads, while for larger beads there is insufficient water flux compensation during spin coating such that beads stick, preventing large scale monolayer formation. The opposite is true for interface coating; larger beads can be used to attain a monolayer more easily (even without the help of surfactant) than smaller beads, which are fragile at the boundaries and have a reduced HCP yield. This difference can be attributed to the surface forces that arise from surface curvature, drawing in nearby beads as the amount of beads increases. Spin coating the 500 nm beads, and interface coating the 1000 nm beads without SDS each reach 90% coverage or greater.
